# A Proper Dietary for Dentists

**Published:** 1916-11

**Authors:** Watson W. Eldridge

**Affiliations:** New York City


### A PROPER DIETARY FOR DENTISTS.

BY WATSON W. ELDRIDGE, M.D., NEW YORK CITY.

The sum and substance of good advice about food is—Eat natural food, not food that has had an artificial preparation. Eat food that is digestible and will call for the exercise of your digestive power. Do not eat food that is predigested or that has had its nutritive principles obliterated by chemical processes designed to give it "appearance" or "refinement."

More than one patient has asked what is the matter with this generation, that it had to "coddle" its stomach through fear of indigestion and other things; why our fathers had so little stomach trouble, and our grandfathers and older progenitors had even less? One patient, unwittingly, expressed a great truth when, after advice not to eat bread and pastries made of white flour, he said, "This is a puny generation. We can't eat this, we can't eat that. Our fathers ate whatever they wished and they had no stomach trouble. The only reason they didn't eat white bread was because they could seldom get it." That is just the point. They could not get things to eat which we are practically forced to buy, because those things had not then been manufactured. When our great grandfather wanted flour he took a sack of whole wheat to the mill and had it ground. He brought back a sack of whole wheat flour, flour that contained all the organic and inorganic principles, required for the proper maintenance of our body metadolism. He did not have a product that had been chemically bleached and treated to make it beautifully white, treated until most of the vitamine* content was killed.

When our great grandmother made rice pudding, it was made with whole natural rice, brown in color, not with the lovely white polished product, which has had the vitamines removed.

A letter from the Office of Home Economics, United States Department of Agriculture, contains this passage regarding polished white rice. "Polishing rice includes the removal of the outer layers of the grain and giving the grain, by mechanical and other means a somewhat burnished appearance. The question of the use of polished or unpolished rice, from the standpoint of health is a matter upon which more or less has been written, particularly because of the relation to disease the use of polished rice is believed to have. The unpolished rice should be used because the branny layers of the rice grain contain minute amounts of substances to which the name 'vitamines' has been applied, which are essential to normal health."

In the old days, people did not eat cold storage eggs with their contents of hydrogen sulphide, cold storage tainted meats, or other forms of chemically preserved and treated foods. They ate natural food, containing all the elements of nutrition. They had to do this, as artificially prepared foods were not then on the market as they are

* The vitamines are portions of the bran parts of grains and other foods, not fully understood chemically, but essential to proper nutrition. They are lost in the preparation of white flour and greatly refined meals.

today. This is why our fathers and grandfathers escaped most of the alimentary troubles that afflict this generation.

Man seems to have lost that inherent ability, called instinct, which enables him to choose the proper foods. Animals do not commit the dietary blunders of which we humans are guilty. We have come to attach more importance to appearance than to quality. Our taste in many instances has become entirely perverted as evidenced by the preference of many people for the taste of white bread, to that of whole wheat or bran bread. Get back into the good old habit of eating bread from whole wheat flour and after a while you will realize how very insipid and tasteless is the white flour product. The same with rice and the products of oats, barley, etc.

Our errors of diet, however, are not confined to those forced upon us by food manufacturers, though these are many and grievous. We, individually, are guilty for many of our gastronomic sins. There is a large class of people in this country, known to most laymen as meat eaters, to most physicians as sufferers from many and various diseases directly traceable to the alimentary tract.

These people have an enormous appetite for meat. They eat it three times a day, and often to the exclusion of most other kinds of foods. They would think they were starved if they had a meal without meat, and as for doing without it for a whole day or a weak—well that would be a crime too horrible to contemplate. Yet their meat consumption is largely responsible for their troubles. I am not a "vegetarian" by any means, but I know the consequences of too much meat and too little of other foods. Meat does not contain enough of the mineral elements required by the body metabolism, and when the diet consists of much meat and little else, the body suffers correspondingly. Meat contains iron, in quantity, and for this reason is good, but only in such amounts as supplies the body with the necessary iron. When this amount is increased, and other food, containing the other necessary minerals, is correspondingly decreased, trouble results. Meat once a day is more that sufficient for any human need and the rest of the daily menu should consist of vegetables, fresh and cooked fruits, whole grain cereals* and whole grain pastries, eggs, and milk. An excessive meat diet is usually found in those suffering from rheumatism, gout, high blood pressure, and the many ailments accompanying constipation. In the latter class of cases, however, I think white bread is equally or more largely responsible, though we find as a rule that excessive meat, white bread and sedentary habit, go hand in hand, pretty uniformly.

Of the other errors for which we individually are responsible, can be mentioned, excessive alcohol, sugar, and stimulating drinks such as tea and coffee. There is no necessity for dwelling on the subject of alcohol. It is already well known to all. Concerning the harm done by an excessive sugar intake we shall first have to consider a phase of its chemistry. Sugar has a tremendous affinity for lime, so much so that whereas water will ordinarily take up one part of lime to the thousand, if a little sugar be added it will take up thirty-five parts of lime, that is, hold it in complete solution. Sugar has a greater affinity for lime than has any other of the compounds taken into the body. The body is composed of about three-fourths water by weight and it takes one part of lime to the thousand to keep that amount of water saturated. Through an excessive sugar intake the water calls for more lime and the affinity between the lime and sugar is such that unless additional lime be found elsewhere, the lack will be supplied from the bones, teeth, etc., leaving these body struc-

* Whole wheat flours differ in characteristics, due to manner of milling, cleanliness, etc. I am able to buy the finest whole wheat bread I have ever eaten from Mr. Fischer, 154 W. 34th St., New York City, in 10 and 15-cent loaves. He tells me it is made from Wheatsworth Whole Wheat Flour.

tures greatly deficient. The bones will become soft and weak and the resistance of the teeth to decay will be lessened. It might be mentioned here that the lime necessary to complete the water saturation will in all probability *not* be "found elsewhere," because lime is one of the very minerals which is so lacking in our diet on account of its absence from processed flour, rice, etc., and which is therefore already deficient in the body.

Coffee and tea are of course greatly abused articles of diet. I can not say food because they are in no sense food, and both contain in greater or lesser amounts a powerful stimulant of the nervous and circulatory systems, namely: caffein. I do not believe that, taken in moderate amounts, they have any very deleterious effect on the body organism, but the trouble is that they are seldom taken in moderate amounts. The coffee drinker, or fiend, usually wants coffee three times a day and two or three cups at a meal. The tea drinker is even worse, having his tea between meals and at bedtime. This constant stimulation to false activity of the systems affected is bound to weaken them in time, and the presence of caffein in the stomach will in addition, retard digestion. These habits, however, like the alcoholic, are so well known and understood that expatiation is useless here.

The evils to be pointed out are those of eating "refined" foods and too much meat.

Bran bread is especially recommended to sufferers from constipation. It not only contains the mineral ingredients and vitamines necessary to body health but the bran flakes themselves are needed to stimulate intestinal peristalsis.

There are of course various diets for various conditions but space here is too limited to go into all of these. There is also much interesting information which has been obtained through recent experimental work relative to individual idiosyncrasy to certain forms of protein foods, which can not be detailed here. For the general guidance of the average person, however, it is safe to say that the dietary should be made up of a small amount of meat once a day or less, or eggs in place of meat, vegetables in variety and season, always fresh when procurable, bread and pastry made from whole wheat or bran flour, raw or cooked fruits, preferably the latter, and milk. Of the vegetables, those which should be preferred in an anti-constipation diet are, spinach, lettuce, endives, asparagus, string beans, kale and greens of all kinds.—*Dental Digest for October.*

